# Facilitators and barriers to implementing and sustaining facility-based stillbirth reviews in India: a qualitative study

**DOI:** 10.1186/s12884-025-07912-6

**Published:** 2025-08-06

**Authors:** Yebeen Ysabelle Boo, Rupanjali Deka, Aliki Christou, Uchenna Gwacham-Anisiobi, Jenny Gong, Monica Lakhanpaul, Jennifer J. Kurinczuk, Manisha Nair

**Affiliations:** 1 National Perinatal Epidemiology Unit, Nuffield Department of Population Health, University of Oxford, Oxford, UK; 2 Srimanta Sankaradeva University of Health Sciences, Assam, India; 3 Institute of Tropical Medicine, Antwerp, Belgium; 4 Population, Policy and Practice Department, UCL Great Ormond Street Institute of Child Health, University College London, London, UK; 5 Department of Biosciences and Bioengineering, Indian Institute of Technology Bombay, Mumbai, India; 6 Public Health Foundation of India (PHFI), New Delhi, India

**Keywords:** Stillbirth review, Facilitators, Barriers, India, MPDSR, COM-B

## Abstract

**Background:**

Stillbirth reviews provide opportunities to improve the quality of maternity care and reduce preventable stillbirths. In India, facility-based stillbirth reviews have been part of the National Health Mission since 2016, yet their implementation has been inconsistent. This study aimed to identify the facilitators and barriers to implementing and sustaining facility-based stillbirth reviews as reported by Indian healthcare professionals.

**Methods:**

A qualitative study was conducted between August to October 2023 using focus group discussions with purposively sampled healthcare professionals from eight hospitals in India, which included those that conduct stillbirth reviews and those that do not. Discussions were analysed using the Framework Method. We applied the capability, opportunity, motivation, and behaviour (COM-B) model to identify facilitators and barriers and used the Behaviour Change Wheel to link these to intervention functions.

**Results:**

Important factors related to capability included record-keeping skills, understanding the importance of documentation, and training on how to conduct effective stillbirth reviews. Under social opportunity, key facilitators were creating a blame-free environment for discussion, ensuring participation from all levels of the health system, and addressing cultural perceptions of stillbirth and post-mortem examinations. Facilitators related to physical opportunity included the presence of clear stillbirth definitions and the collection of minimum stillbirth data. Reflective motivation, specifically, the beliefs in the benefits of stillbirth reviews for both the facility and the community, was a notable factor in the willingness of healthcare professionals to implement and sustain stillbirth reviews despite system-level barriers.

**Conclusions:**

Strengthening the implementation and sustainability of facility-based stillbirth reviews in India requires targeted, practical interventions. Creating a blame-free environment can be supported through workshops emphasising the learning purpose of reviews, having neutral facilitators to guide discussions, and asking participants to sign a code of conduct ‘charter’ pledging their commitment to providing a safe environment for all panel members and the community, incorporating this into local stillbirth review guidelines. Clear national guidelines on stillbirth definitions, combined with training on accurate data collection and reporting, will improve consistency. Regular training and mentoring should focus on building healthcare professionals’ capability in record-keeping and effective review practices. Strengthening information technology infrastructure and providing protected time for reviews will address workload challenges. Additionally, fostering motivation through peer-led discussions and sharing positive outcomes can encourage continued commitment to stillbirth reviews.

**Supplementary Information:**

The online version contains supplementary material available at 10.1186/s12884-025-07912-6.

## Background

The fundamental aim of a stillbirth review is to “support objective, robust and standardised review to provide answers for bereaved parents about why their baby died” [1] and to “ensure local and national learning to improve care and ultimately prevent future deaths” [1]. Facility-level stillbirth review is especially critical for assessing the incidence, causes, risk factors and quality of maternity care to guide evidence-based interventions for reducing stillbirths.

Despite global efforts, stillbirth remains a significant global health challenge. In 2021, a staggering 1.9 million babies were stillborn worldwide, with India accounting for 286,482 deaths - the highest absolute number of stillbirths recorded in any country globally, consistently observed over the past two decades [2–4]. While India has made progress in reducing stillbirth rates, achieving a decline from 29.8 per 1,000 total births in 2000 to 12.2 per 1,000 in 2021, the burden remains substantial [3]. Recognising this challenge, India launched the India Newborn Action Plan with the goal of reducing the stillbirth rate to below 10 per 1,000 total births by 2030 [5].

To support this, the National Health Mission introduced operational guidelines for establishing the Sentinel Stillbirth Surveillance System in 2016, providing a framework for standardising facility-based stillbirth reviews [6]. These guidelines were developed by the World Health Organization (WHO) Country Office for India in collaboration with India’s Ministry of Health and Family Welfare. They form part of the broader Maternal and Perinatal Death Surveillance and Review (MPDSR) system, which aims to improve maternal and perinatal outcomes through systematic identification, notification, and review of deaths. The MPDSR system aligns with the WHO guidelines, Making Every Baby Count: audit and review of stillbirths and neonatal deaths [7], also published in 2016, which provide practical guidance for conducting stillbirth and neonatal death reviews and ensuring that findings are used to improve care.

While stillbirth reviews have the potential to improve the quality of maternity care and reduce stillbirth, their implementation is often inconsistent [8–10]. We conducted a qualitative study with the aim of identifying facilitators and barriers to implementing and sustaining facility-based stillbirth reviews in India.

## Methods

### Study design

This study was a qualitative study in eight hospitals. This study was conducted within the Maternal and Perinatal Health Research collaboration, India (MaatHRI), an Indian collaborative research platform established in 2018 [11].

### Data collection

Eight focus group discussions (FGDs) (one per hospital; three hospitals that conduct stillbirth reviews and five that do not) were conducted between August and October 2023 to understand their knowledge and perceptions of stillbirth reviews. FGDs were chosen as they facilitate discussions that uncover attitudes, feelings, beliefs, experiences, and reactions through the social dynamics and interaction that may not emerge in 1:1 interviews [12–14]. Prior to fieldwork, YYB undertook a qualitative research methods training. At the point of data collection, YYB was a female Doctor of Philosophy (DPhil) student [15] and was known by some FGD participants through prior attendance at MaatHRI meetings, as several participants were MaatHRI collaborators. YYB critically reflected on the ethical considerations, power dynamics, and positionality inherent in conducting research with participants to whom she had pre-existing relationships; these reflections, including strategies to mitigate potential biases and maintain confidentiality, are elaborated in Appendix A. The reasons for conducting the research were provided to all participants in the participant information sheets and reminded again before gaining informed consent.

The study received ethics approval from a subcommittee of the University of Oxford Tropical Research Ethics Committee (OxTREC) (reference number: 539−23). Considering that this was a DPhil research, an ethics approval waiver was granted from local participating hospitals. Written informed consent was obtained from all participants using a standard consent form prior to the FGDs.

Five FGDs were conducted in person, four in a room at the hospital and one in a nearby cafe, while three were conducted online to provide flexibility and accommodate participants’ availability. For one of the FGDs that was conducted in a nearby cafe, the cafe was a hired venue, selected and reserved by one of the participating hospitals specifically for the FGD to ensure privacy and provide a safe environment for the discussions of a sensitive topic. A topic guide was used to facilitate the FGDs. Topic guides were developed based on the study objectives and literature. They were tailored for hospitals based on whether or not they conduct stillbirth reviews (Appendix B and Appendix C).

FGDs were conducted in English as we were informed that English is the preferred language spoken by participants. English is also the standard language of medical training and communication in the participating hospitals. Participants came from diverse linguistic backgrounds and confirmed their comfort with English, which was evident in their engagement during discussions. Each FGD took about 30 to 60 min and was audio recorded with informed consent from the participants. Discussions ended naturally, without being limited by time. The facilitator (YYB) used the topic guide to ensure all key areas were addressed and provided prompts as needed. At the end of the FGD, participants indicated they had shared all they wished to before concluding, suggesting that data saturation had been reached within the focus group.

### Participant selection

All secondary and tertiary MaatHRI hospitals were approached with information about the study’s aim and objectives. Eight MaatHRI hospitals expressed interest, and healthcare professionals (HCPs) involved in maternity care or the stillbirth review process were informed about the study through participant information sheets distributed via email to MaatHRI collaborators, as well as through verbal communication during online MaatHRI meetings. Some MaatHRI hospitals have established stillbirth review processes, while others do not. FGDs were conducted in both hospital types to capture common and different themes, ensuring maximum variation of experiences. Purposive sampling was used to recruit six to eight participants per FGD, prioritising review panel members in hospitals with stillbirth review processes and key maternity care stakeholders in those without. The MaatHRI Project Manager (RD) and MaatHRI collaborators facilitated recruitment. Participation was voluntary, and participants were reminded at the start of the FGD that they were free to ask the facilitator to pause or stop the FGD at any time as per the distress policy. Two participants (one per FGD, across two FGDs) dropped out on the day, one because the participant could not be released from clinical duties and one because the participant did not wish to provide written consent.

### Data analysis

We used the Framework Method to analyse data [16, 17], following Gale et al.’s guide [18]: (i) transcription, (ii) familiarisation with the data, (iii) coding, (iv) developing an analytical framework, (v) applying the framework, (vi) charting data into the framework matrix, and (vii) interpreting the data. Audio recordings from FGDs were transcribed verbatim by an approved transcription company, with quality checks conducted by YYB. Three researchers (YYB, AC, JG) independently coded a subset of transcripts to develop an analytical framework, with discrepancies resolved through discussion. The framework was then applied to the remaining transcripts using NVivo 14 [19], allowing for efficient coding and thematic analysis. Codes were grouped into themes using the capability, opportunity, motivation, and behaviour (COM-B) model, and the final framework comprised 40 codes across five themes. Charting data into a framework matrix enabled systematic comparison, and interpretation, considered the implications for implementation within hospitals and the wider Indian context.

### Theoretical framework

Implementing stillbirth reviews in hospitals requires a system-wide change addressing facilitators and barriers to implementing and sustaining stillbirth reviews at all levels (community, HCPs, facility and policy). Similar to Willcox et al.’s [9] systematic review, this study utilised the COM-B model to develop themes from identified facilitators and barriers to implementing and sustaining facility-based stillbirth reviews in India from the collected qualitative data and the Behaviour Change Wheel to identify actionable interventions by linking these facilitators and barriers to the Behaviour Change Wheel intervention functions. The definitions for the COM-B domains used in this study were also adapted from Willcox et al. [9]., along with the original definition used for broader policy research by Michie et al. [20, 21] (Table 1).

**Table 1:** Definitions for the capability, opportunity, motivation and behaviour (COM-B) domains [20, 21]

• *Capabilities*: the knowledge and skills required at the community, facility, and policy levels to implement and sustain stillbirth reviews in hospitals. • *Opportunities*: all external factors (physical and social) at the community, facility, and policy levels needed to implement and sustain stillbirth reviews in the hospitals. This domain was sub-categorised into physical opportunities and social opportunities. o Physical: environmental context and resources such as time, data, location and resources used in stillbirth reviews o Social: social influences such as cultural norms and social cues within the hospital or wider Indian context • *Motivation*: all factors that energise or influence the internal processes at the community, facility, and policy levels to implement and sustain stillbirth reviews in their hospitals. This domain was sub-categorised into automatic motivation and reflective motivation. o Automatic: habitual processes and emotional responses such as desires, impulses and inhibitions o Reflective: conscious, analytical decision-making such as making plans and evaluating things that have already happened	

### Reporting

To protect anonymity and confidentiality, we have not disclosed which hospitals conducted stillbirth reviews, nor included identifiable participants or site-level details in the results. Validation and dissemination workshops were held with MaatHRI collaborators to review our reporting approach and interpretations. Collaborators were given the opportunity to review the results and discussion to ensure contextual accuracy while maintaining confidentiality.

## Results

**Fig. 1:**
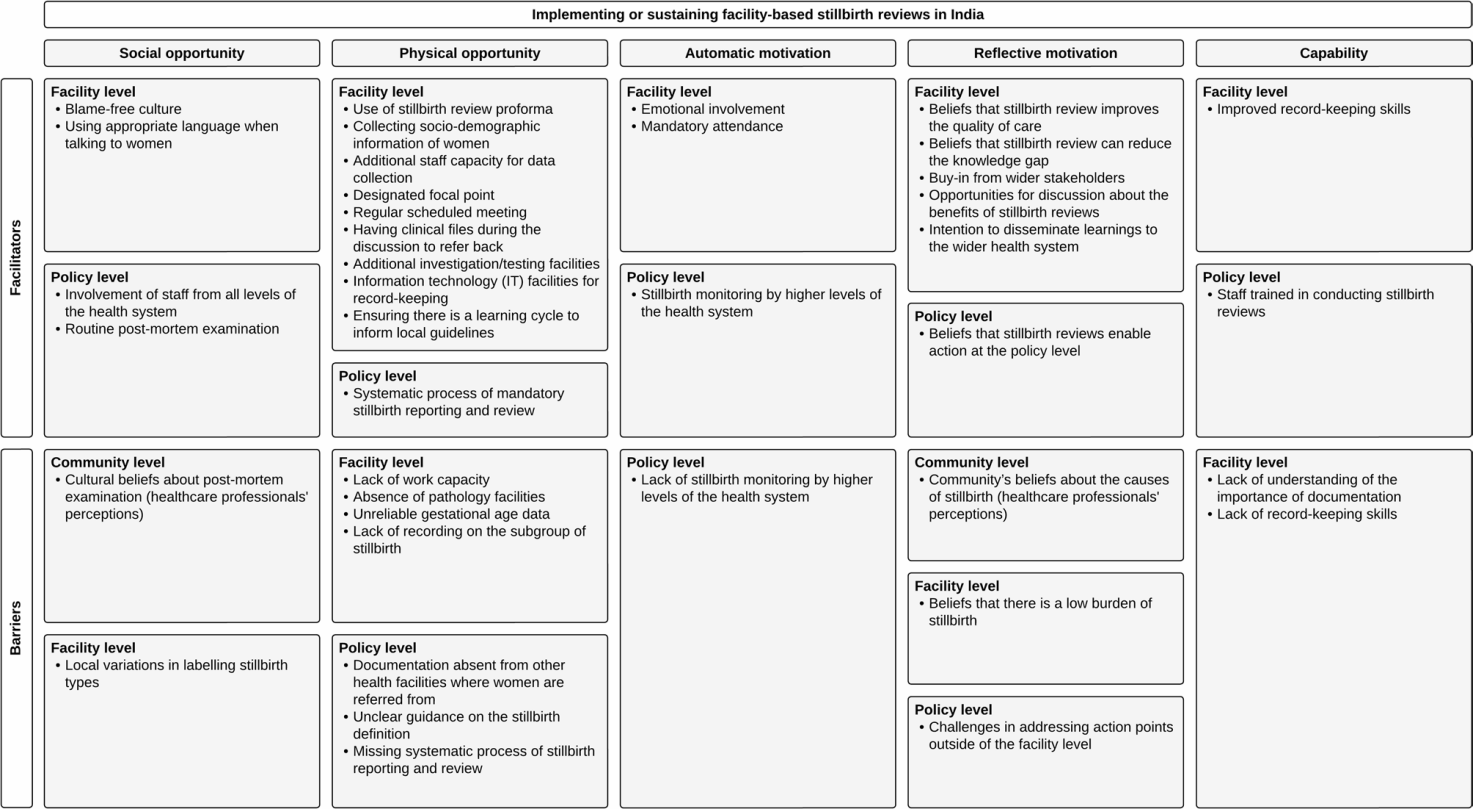
Coding tree representing the themes and codes that emerged as facilitators and barriers

The framework analysis of FGDs identified facilitators and barriers influencing the implementation and sustainability of stillbirth reviews across eight hospitals. These findings were categorised under COM-B domains at the community, facility, and policy levels. Figure 1 presents the coding tree, summarising themes and codes from the analysis.

For consistency, the three hospitals conducting stillbirth reviews are referred to as ‘Review hospitals’, while the five that do not are ‘Non-review hospitals’. Participant statements and perspectives are attributed to their hospitals, reflecting their collective voices. For example, a quote from a hospital (FGD identification number: 2) not conducting stillbirth reviews is labelled *Non-review.2 Hospital*.

Table 2 summarises the characteristics of the participating hospitals and FGD participants’ demographics, including aggregated figures for total births in 2023 and estimated stillbirth rates (per 1,000 total births), derived from data collected through the MaatHRI cohort study [22], to provide context on the level of care and stillbirth burden. The Non-review hospitals were on an average catering to a much larger number of births and had a higher stillbirth rate compared with the Review hospitals.

**Table 2:** Characteristics of hospitals that participated in the study and demographic characteristics of the focus group discussion participants

	Stillbirth review hospitals (*N* = 3)	No stillbirth review hospitals (*N* = 5)
Hospital type		
Tertiary - government	1	4
Tertiary - private	1	1
Secondary - government	1	0
Total births (2023)	12,261	44,599
Average number of births (2023)	4,087	8,920
Stillbirth rate (per 1,000 total births)	10.7	21.8
Focus group discussion participants (N)	20	40
Method of discussion		
Online	1	2
In person	2	3
Job role		
Consultant doctor – obstetrics & gynaecology	5	8
Senior resident – obstetrics & gynaecology	0	2
Postgraduate student – obstetrics & gynaecology	7	1
Consultant doctor – paediatrics	0	6
Consultant doctor – community health	0	1
Consultant doctor – pathology	0	1
Administration/superintendent	1	2
Nurse manager	3	5
Staff nurse	4	11
National Health Mission Community Health Officer	0	3
Gender		
Female	19	25
Male	1	15
Age group		
Under 30 years	10	9
30–49 years	7	23
50 years and over	3	8

The section below presents facilitators and barriers to implementing and sustaining facility-based stillbirth reviews, as reported by HCPs. Findings are organised under theme headings based on the COM-B model, with minor themes presented as sub-themes within each domain. To present the themes coherently, reporting was ordered based on the number of issues raised by the participants within each theme or the number of sub-themes generated with each theme (starting with ‘opportunity’) instead of ordering as per the COM-B acronym, which begins with ‘capability’. Direct quotes from FGDs are included to illustrate key perspectives.

### Social opportunity

#### Opportunities to participate without fear of blame

Fostering a blame-free culture at the facility level was reported as a facilitator for enabling constructive discussions during stillbirth reviews. One hospital from the Non-review group noted that stillbirth reviews should be viewed as opportunities for systemic learning and improvement.

“And the review process should be – it should never be a fault-finding one. This is wrong. It should not be. It should be constructive so that we can save the next baby.” (Non-review.2 Hospital)

This hospital noted that using appropriate language with parents about stillbirth is important for collecting additional information to aid the review process. The hospital emphasised that collecting information non-accusatorily reduces social pressure in doctor-patient interactions, as ‘scolding’ language may lead patients to withhold important information.

“How we get the information from the parents or maybe the mother. (…) yes, that can only be done if you do not accuse them. If you are scolding, then they will try to hide.” (Non-review.2 Hospital)

These reflections show how social cues, cultural expectations and interpersonal norms, within clinical teams and between HCPs and parents, shape conditions for open, blame-free dialogue, highlighting social over structural influence.

#### Opportunities for staff from all levels of the health system to participate

Two hospitals recognised that involving staff at all levels in stillbirth reviews, not just doctors, improves the quality of the review process. A Review hospital noted that including various roles, such as administrative staff and nurses, improves learning by incorporating diverse perspectives. A Non-review hospital noted that engaging community health workers providing antenatal care could offer a more complete picture of stillbirth. This approach is more valuable than relying solely on antenatal care notes and aligns with the current maternal mortality meetings process in Indian hospitals.

“But only one part is we need those who have provided her [women’s] antenatal care. (…) To get a complete review, and that is the way we are doing in maternal mortality.” (Non-review.2 Hospital)

#### Navigating cultural challenges in obtaining consent for post-mortem examinations

Cultural beliefs about post-mortem examinations hinder accurate identification of stillbirth causes. A Review hospital reported that obtaining consent is particularly challenging in India due to prevailing cultural beliefs, often upheld by family members like grandparents. The hospital felt this challenge would continue despite government regulations or designated post-mortem units.

“It will be very difficult because of the cultural belief that’s also important in this part; they will not allow, even if the government brings a rule or we have a post-mortem unit (…) The grandmother especially, rather than the parents [laughs], will disagree to the examination.” (Review.3 Hospital)

One hospital in the Non-review group suggested that government authorities could facilitate routine post-mortem examinations with an opt-out option to address this challenge, similar to their routine HIV screening programme.

“Yes, I think the resources that we need is if we have a kind of hospital authority making it routine autopsy for all stillbirths. (…) It’s like HIV [human immunodeficiency virus] screening, you know, opt-out.” (Non-review.1 Hospital)

#### Understanding local variations in labelling stillbirth types

Two of eight hospitals labelled stillbirths before labour as ‘antepartum’ and ‘old’, while the other six referred to them as ‘intrauterine deaths (IUDs)’. Moreover, two hospitals noted that only intrapartum stillbirths are termed ‘stillbirths’.

“We find out if there is an absence of FHS [fetal heart sound]. Then we consider after a scan or a proper scan, we diagnose there is an intrauterine fetal demise. So, we take it as an IUD [intrauterine death]. Whereas for a stillbirth, we consider, during the process of delivery, if the FHS was still present during the process of delivery, so if the child comes out - what to say - there was no response, or there is an absence of heart rate so to say, so we take those as stillbirths.” (Review.3 Hospital)

### Physical opportunity

#### Using standard proforma to collect stillbirth data

Two hospitals from the Non-review group highlighted the need for a standard proforma for stillbirth reviews to facilitate systematic data collection. One hospital mentioned that proper formatting and record-keeping are challenges, and having a proforma would help standardise the collection of data and aid in the review process. Both hospital types referenced the use of proformas in maternal mortality reviews as an example to follow in stillbirth review process.

“If it is like whatever we have in maternal death review. (…) We have a standard proforma to be filled up.” (Non-review.2 Hospital)

One hospital that currently employs a proforma for its stillbirth review reported that it helps gather consistent information about stillbirths, such as admission details, time of birth, and whether the case was booked or unbooked. However, this hospital highlighted that it is still important to have clinical files available during review meetings in case details are missed on the proforma.

“We have a file where we have a questionnaire as per the hospital, where we find out the causes, and the questionnaire, for example, what time the admission was done, what time she delivered, was she a booked case or an unbooked case? (…) During the discussion actually, we should have the patient’s file with us, the patient’s clinical file from when she was admitted; it should be with us. And all the clinical notes from page one to the last page with all investigations report we have to review that.” (Review.3 Hospital)

#### Minimum stillbirth data required for reviews

One hospital from the Non-review group emphasised the importance of collecting socio-demographic information as part of stillbirth data. They suggested that every woman admitted to the labour room should undergo a social survey to gather this type of information, which is often missing and difficult to follow up once a woman is discharged.

“Every patient or every mother who is admitted in labour room should have a social survey.” (Non-review.4 Hospital)

Collecting data on types of stillbirth was another area that hospitals wanted to improve in their stillbirth data collection since there is currently a lack of recording of such information.

“It’s quite hard to collect the [stillbirth] data. We just take the numbers.” (Non-review.1 Hospital)

A major barrier in gathering minimum data for stillbirth reviews is the frequent lack of documentation from health facilities which the woman visited prior to arriving at the facility where she gave birth. Two Review hospitals reported that the absence of clinical notes during pregnancy hinders the process of action planning. One Review hospital stated that despite documentation challenges, they aim to “try to find” any available data, recognising that even minimal stillbirth data can significantly improve care for women.

“Those cases which have already come with a stillbirth from outside, in those cases it’s a little bit difficult to get into the very minute details because we won’t have so much data. But whatever data we have, accordingly, we try to find it.” (Review.3 Hospital)

Non-review hospitals also identified this barrier, particularly regarding the lack of recorded fetal heart rates, making it challenging to determine if the stillbirth occurred before or during labour. One Non-review hospital stated that conducting a stillbirth review was virtually impossible due to the lack of information about the active labour phase and delays in transfer. This challenge not only significantly limited their ability to review stillbirths but also led to frustration within the system.

“Maybe [we can discuss stillbirth]. But we would not have done it very elaborately because it is not like we were monitoring the labour throughout. We would have never monitored the person. (…) We will not be able to [conduct a review], because it is difficult for us to get all the information about the patient.” (Non-review.4 Hospital)

#### Availability of information technology (IT) facilities for record-keeping

Two hospitals from the Review group identified IT facilities as a key facilitator for improving record-keeping. They noted that managing records on paper is prone to loss and often results in incomplete data during stillbirth reviews. Another Review hospital highlighted the benefits of their electronic medical record (EMR) system in managing data efficiently and reducing the risk of losing important information.

“We have an EMR [electronic medical record] system from the obstetrics department; all the data is collected and saved. (…) So, I think in our hospital, this is up to the mark now; we don’t have any problem regarding managing data.” (Review.3 Hospital)

In contrast, Non-review hospitals did not identify the availability of IT facilities as a facilitator. Instead, they were more focused on ensuring minimum stillbirth data is collected using standard proformas to facilitate a comprehensive data collection process.

#### Unclear guidance on the stillbirth definition

One non-review hospital reported that an unclear stillbirth definition leads to deprioritisation of stillbirths, with confusion surrounding the 24 versus 28 weeks gestation cut-off for stillbirth. Another hospital echoed these concerns, noting potential under or over-reporting due to lack of clarity. They urged the Indian government to define stillbirths clearly for better guidance in practice.

“What is a particular stance of the government of India, that is our importance. Whatever government decides – they say we take 28, we take 28.” (Non-review.2 Hospital)

#### Systematic process of mandatory stillbirth reporting and review

All three Review hospitals emphasised the importance of a systematic process for stillbirth reporting and review. They explained this approach ensures that all stillbirths are counted and reviewed thoroughly. The hospitals also reported that the government-mandated reporting processes support this systematic approach by requiring hospitals to report stillbirth statistics monthly. They explained that this mandatory obligation ensures that all stillbirths are counted and considered as potential stillbirths for review.

“It [stillbirth report] will be handed over to the concerned person who is sending the report every day to the CMO [Chief Medical Officer] office. (…) No stillbirth is missed.” (Review.1 Hospital)

A non-review hospital reports monthly stillbirth statistics, indicated that the policy-level reporting requirement could be integrated into their approach for identifying eligible stillbirths. Another Non-review hospital described an ideal stillbirth review process, including prompt notification of stillbirths, immediate registration for reporting, and regular reviews of registered stillbirths.

“I think the process will be like recording, then immediately registering them, and then have a monthly or two-monthly review of the cases.” (Non-review.2 Hospital)

#### Challenges in high workload

Both hospital types highlighted challenges related to staff capacity impacting their ability to organise and conduct stillbirth reviews. For instance, some hospitals struggle with record-keeping due to the high demands on their staff involved in direct clinical care, and sometimes, this leads to incomplete or missing routine health records, impacting stillbirth reviews.

“No, I am saying that record keeping can sometimes become difficult because we have to do delivery also, no? So sometimes record keeping becomes difficult for us. That’s why some records may be lacking, won’t be there.” (Review.2 Hospital)

Two hospitals emphasised that appointing additional staff could help with the burden associated with stillbirth data collection and meetings, given the staff shortages and workload. However, hospitals understood that this may not be possible without funding and resources and that they will still attempt to review stillbirths thoroughly with existing data.

“Unless we have a team, which can individually go to their home and get the output from the medical and the primary health centres. But you have to do whatever you can with the data you have if you can.” (Non-review.4 Hospital)

#### Scheduling stillbirth review meetings

All three hospitals in the Review group agreed that having a regularly scheduled meeting for stillbirth reviews is an important facilitator. One hospital highlighted that having a specific day each month for the stillbirth review improves attendance.

“And if a particular day of the month is scheduled for a review meeting, then I think it will be better. Then one that day, we can keep more staff, so that’s how they can come for the meeting.” (Review.2 Hospital)

Additionally, a Review hospital noted that having a designated focal point is crucial for ensuring the review process is integrated into daily practice.

“So the nodal officer is there, so it is my responsibility. […] I have to look after the stillbirth if this happens, I make sure that there is a stillbirth review.” (Review.1 Hospital)

#### Additional investigation/testing facilities to improve the completeness of stillbirth data

One Review hospital reported a lack of facilities for conducting post-mortem examinations, additional investigations or testing, such as pathology laboratories, which are necessary for understanding the causes of stillbirth.

“We don’t have a system of any post-mortem for the fetus. (…) maybe in higher centres they will, and they will evaluate the fetus, do the post-mortem of the fetus, take specimens.” (Review.3 Hospital)

In contrast, a Non-review hospital highlighted that the presence of a pathology department enables them to perform additional investigations, including fetal post-mortem examination, to determine the causes of stillbirth.

“We do investigate, and we do some special tests for the patient to find out particular causes in them. At present, a study is being undertaken by our postgraduate student. An autopsy of stillbirths.” (Non-review.1 Hospital)

#### Using a reliable method to determine accurate gestational age

One Non-review hospital identified unreliable gestational age data as a barrier to stillbirth review, noting that calculating gestational age by the last menstrual period (LMP) is often unreliable. While sonography, which is required for accurately determining gestational age, was recognised as a potential facilitator, the hospital reported that some women may not have received an ultrasound examination.

“I think another issue is like this LMP [last menstrual period]. I mean, this dating of pregnancy by their history is also very, very – not very reliable. (…) The majority group do not go for sonography.” (Non-review.2 Hospital)

#### Ensuring there is a learning cycle to inform local guidelines

One Review hospital emphasised that review findings should be documented and inform formal local clinical guidelines rather than being communicated solely through verbal discussions. This hospital noted that currently, they only share insights from stillbirth reviews verbally, and it often leads to varied clinical practices based on individual experiences rather than evidence-based guidelines. This variability in treatment approaches was identified as a challenge, as it may result in inconsistent care.

“It is only verbal [dissemination of learnings]. There I think that our institute is lacking. (…)The protocol, the SOPs [standard operating procedures] should be there of a particular institute because everyone is giving us the guidelines [on how to treat the women].”

### Automatic motivation

#### Emotional involvement

A Review hospital described that the emotional impact of stillbirths on HCPs motivates their participation in stillbirth reviews. This hospital described that the tragedy of stillbirth often leads the staff to be emotionally involved with the family and feel a strong personal commitment to actively engage in reviews.

“Actually, where that’s a tragic incident, no? (…) They are self-motivated to come and follow up these things.” (Review.2 Hospital).

#### Mandatory attendance to stillbirth reviews

Mandatory attendance emerged as an automatic motivator for certain job roles within hospitals to participate in stillbirth reviews. At a Review hospital, for example, postgraduate students who are medical doctors pursuing advanced training in a specific specialty (referred to as postgraduate students) were required to attend stillbirth reviews as part of their internal assessments.

“We have students who are bound to attend, their attendance is marked, and their internal assessment is based on that, so the choice of not attending is not there! [laughs]” (Review.1 Hospital).

#### Stillbirth monitoring by higher levels of the health system

Stillbirth monitoring by higher levels of the health system, such as the National Health Mission, was described as a facilitator that stems from reinforcement, which prompts hospitals to carry out their own stillbirth reviews to improve their stillbirth reporting to the higher levels of the health system.

“That data will be compiled at the national level, and then the protocols and the guidelines which we get from the NHM [National Health Mission], that is from that data. (…) The stillbirth reviews from here go in there [NHM].” (Review.1 Hospital).

A Non-review hospital noted that where there is a lack of stillbirth monitoring at higher levels, stillbirths are often unaccounted for by both obstetricians and paediatricians, and reviews would not be initiated by either department in the hospital. This lack of monitoring at higher levels of the health system creates a barrier to conducting stillbirth reviews in India, as there are no incentives or negative consequences for hospitals if they do not report, review, or analyse stillbirths that occur during their care.

“So these [stilbirths] are the, you know, the deaths of the babies which are going unmonitored, and it’s really a big issue for us to see why there’s that many. Because we [obstetricians] are not taking accountability, the neonatologist or the paediatricians are not taking accountability of the stillbirth.” (Non-review.3 Hospital).

### Reflective motivation

#### Beliefs that stillbirth reviews enable action

A review hospital discussed that knowing a stillbirth review improves the quality of future care is a facilitator to sustaining stillbirth reviews. This hospital explained that recommendations from the stillbirth reviews are taken on board by the staff, and they observe positive changes in practices and infrastructure afterwards.

“I think they take inputs from us, from everyone involved in the labour room team and, based on those inputs, if there are some changes made in terms of infrastructure or the staff or duty roster.” (Review.1 Hospital).

Some hospitals anticipated that stillbirth reviews would prompt policy actions. They believed findings could influence policymakers to prioritise interventions to reduce stillbirths. Highlighting deficiencies in resources or staff skills through sharing learnings could encourage policymakers to address these barriers.

“Local government staff will then [once stillbirth review findings are disseminated] be ready to put more attention on stillbirth prevention.” (Non-review.4 Hospital).

#### Beliefs that there is a low burden of stillbirth

A hospital reported reviewing all stillbirths due to a perceived low burden in their catchment area. This belief, regardless of whether this was really true, was seen as a facilitator because this hospital remained positive about being capable of reviewing all stillbirths and motivation to conduct thorough reviews for each stillbirth to improve their current practice.

“We don’t have many stillbirths, so it’s [reviewing all cases] not a problem for us.” (Review.2 Hospital).

#### Community’s beliefs about the causes of stillbirth (perceived by HCPs)

A Review hospital expressed their concerns regarding the influence of cultural beliefs on stillbirth inquiry in the communities. They explained that the community holds strong cultural beliefs that stillbirth occurrence is spiritual, and this perspective often leads them to be reluctant to pursue further investigations or interventions. This challenge was identified as a demotivating factor for sustaining the stillbirth review process, as hospitals felt they might have to act against community beliefs when investigating the underlying causes of stillbirths and implementing preventive measures.

“The problem is that in our culture, if there are any stillbirths or even, for example, it was a sudden IUD [intrauterine death], it’s their religious belief that it’s God’s doing, so they just accept it like that. (…) So that is one hurdle, so for us to go further ahead and find out the cause, especially the genetic and the chromosomal causes, it will be quite difficult.” (Review.3 Hospital).

#### Beliefs that stillbirth review can reduce the knowledge gap

Hospitals’ beliefs that stillbirth reviews can reduce the knowledge gap at both the facility level and within the wider health system were reported as important facilitators. One Non-review hospital highlighted that staff will be motivated to participate because the evidence presented from stillbirth reviews will provide meaningful feedback to doctors and increase overall awareness of common issues related to causes of stillbirth. A review hospital already recognised the benefits of stillbirth reviews in generating knowledge for the broader clinical team.

“Most of the stillbirths, the unusual ones or the ones which were a little bit controversial, we remember. We remember all those cases vividly. (…) It’s for their learning. If they encounter such kinds of patients, they can apply this knowledge. It’s lifelong learning.” (Review.1 Hospital).

#### Buy-in from wider stakeholders

Both review and non-review hospitals emphasised that securing buy-in from key stakeholders is crucial to initiating and sustaining stillbirth reviews within the hospital. A Non-review hospital pointed out that getting buy-in from diverse departments at the facility level, such as pathology and forensic medicine, is a priority. They acknowledged that this can be challenging, as not everyone may be receptive to the idea of a stillbirth review process, and overcoming resistance would be a significant challenge.

“Pathway required from the pathology department, forensic department, all of the departments. I think the biggest challenge will be that, because some people, they don’t like that. Most of them. Most of the people are opposed to it.” (Non-review.1 Hospital).

#### Opportunities for discussion about the benefits of stillbirth reviews

At the end of the FGD, a Non-review hospital highlighted that FGD was an important ‘sensitisation’ exercise to think about the stillbirth review process and that the discussion from the FGD boosted their motivation to implement stillbirth reviews within their hospital. Prior to the FGD, the hospital had recognised the importance of conducting these reviews but had struggled to initiate the process due to various constraints.

“I just want to tell you – this was a good sensitisation procedure what you have come and told us about. This [facility-based stillbirth review] has to be done. It’s a very good way of sensitising us that we can put this in our plate of review meetings, if possible.” (Non-review.1 Hospital).

### Capability

#### Record-keeping skills and Understanding the importance of Documentation

Two hospitals (a review hospital and a Non-review hospital) emphasised the importance of training postgraduate students in record-keeping skills, noting that this is a mandatory responsibility for doctors rather than an optional task. The hospital explained that providing record-keeping training is an important facilitator for stillbirth reviews because the training can improve the completeness of routine health records, which is essential for accurately filling out stillbirth review proforma.

“The juniors, every discussion we have, we stress the point to them that documentation, documentation, documentation (…) even if you don’t want to do it, you will have to do it.” (Review.3 Hospital).

#### Staff trained in how to conduct effective stillbirth reviews

Finally, a Non-review hospital recognised that staff must be trained before implementing stillbirth reviews. They pointed out that if the staff are not trained, they may not be able to identify relevant causes or suboptimal care factors associated with stillbirth or lead meaningful discussions to develop appropriate recommendations of actions for implementation to reduce future stillbirths.

“The person who will be leading the review, he or she should be trained. That is another important issue because unless he’s trained, then he may not be able to review or may not be able to review or lead that discussion on the review process.” (Non-review.2 Hospital).

### Actionable interventions

Table 3 presents proposed actionable interventions designed to upscale facilitators and address barriers reported by Indian HCPs. These interventions were mapped from the COM-B model and the Behaviour Change Wheel intervention functions, offering targeted and theory-based strategies to improve social and physical opportunities, automatic and reflective motivation, and capability that influence the implementation and sustainability of facility-based stillbirth reviews. For instance, within the social opportunity domain, ‘opportunities to participate without fear of blame’ is suggested to be addressed through interventions such as regular workshops to emphasise the purpose of stillbirth reviews as a tool for learning and improvement rather than blame. Additionally, the presence of a neutral meeting facilitator is recommended to ensure meeting principles, such as a blame-free culture, are communicated and upheld during review meetings.

**Table 3:** Proposed actionable interventions for facilitators and barriers using the behaviour change wheel intervention function

COM-B domain	Facilitator/barrier reported by Indian HCPs	Proposed actionable interventions identified using the Behaviour Change Wheel intervention function
Social opportunity	• Opportunities to participate without fear of blame • Opportunities for staff from all levels of the health system to participate • Navigating cultural challenges in obtaining consent for post-mortem examinations • Understanding local variations in labelling stillbirth types	*Education* • Increase the HCPs’ and community’s knowledge about what post-mortem examination entails (e.g. different types of post-mortem examinations) • Organise regular workshops that emphasise the learning purpose of stillbirth reviews, helping all levels of the health system to understand that the goal is to improve the quality of care rather than blame or discipline individuals *Enablement* • Have a neutral meeting facilitator who will communicate and enforce meeting principles during the stillbirth review meeting • Have open communication with the HCPs on which labels are most appropriate to categorise different types of stillbirth ensuring they align with accepted international definitions and discuss potential positive or negative implications for having local variations • Invite staff from different levels of the health system to the stillbirth review meeting if they are involved in women’s care *Environmental restructuring* • Create terms of reference for review panel that includes purpose of the review, authority of the panel, panel membership, duties and responsibilities of the panel, and details of the procedural issues (e.g. frequency of meetings, quorum, completion and distribution of meeting minutes and action points timeline) • Ask current and potential participants (including those who collect data) to sign a code of conduct ‘charter’ pledging their commitment to providing a safe environment for all panel members and the community and incorporate this ‘charter’ into the local stillbirth review guidelines • Develop national guidelines for labelling and defining stillbirth types while ensuring consistency with national or international definitions *Modelling* • Encourage senior staff (e.g. consultants, superintendent) to demonstrate a blame-free, supportive culture where review discussion focuses on system-level issues and not on individual performance *Persuasion* • Develop culturally sensitive communication materials (e.g. pamphlets and videos) that address common concerns about post-mortem examinations, emphasising the benefits of system change to improve the quality of care in women • Include testimonials or examples of how stillbirth reviews have led to positive changes in the system (e.g. improvements in monitoring protocols or infrastructure) *Restriction* • Explore with the community whether an opt-out consent system for post-mortem examinations could work in the Indian context *Training* • Provide opportunities where the HCPs can practice how to communicate with the family about obtaining consent for post-mortem examinations • Provide training for the HCPs on national and international definitions of stillbirth and how these can be applied to ensure consistency in stillbirth reporting • Provide training for pathologists on minimally invasive post-mortem techniques and imaging, allowing parents the option to choose different levels of post-mortem examination rather than being limited to a complete post-mortem (the traditional method involving surgical incisions)
Physical opportunity	• Using standard proforma to collect stillbirth data • Minimum stillbirth data required for reviews • Availability of IT facilities for record-keeping • Unclear guidance on the stillbirth definition • Systematic process of mandatory stillbirth reporting and review • Challenges in high workload • Scheduling stillbirth review meetings • Additional investigation/testing facilities to improve the completeness of stillbirth data • Using a reliable method to determine accurate gestational age • Ensuring there is a learning cycle to inform local guidelines	*Education* • Increase the community’s knowledge about what ultrasound entails *Enablement* • Create protected time for staff to focus on stillbirth reviews without competing with other work demands • Designate a team for documenting and formalising stillbirth review findings into local clinical guidelines and other action points • Establish partnerships between hospitals with different investigation and testing facilities, creating a referral and transfer system that allows hospitals without these resources to access them as needed • Provide additional resources such as extra staff to assist with data collection and organising review meetings *Environmental restructuring* • Clearly define and assign specific job roles within the hospital to be accountable for counting and reporting stillbirths • Develop a standard proforma for the local use from the existing national or international guidelines that would help with data collection for stillbirth review • Develop and disseminate clear, standardised definition of stillbirth to be documented in local and national clinical guidelines, including criteria for different subgroups of stillbirth to ensure consistency across different levels of health system • Ensure hospitals are equipped with the necessary ultrasound equipment • Establish a standardised referral and information-sharing system between different levels of health facilities that ensures the transfer of clinical documentation when women are referred • Develop protocols that prompt the use of sonography in all women, and especially in cases where gestational age is uncertain • Include sections of socio-demographic and clinical data that must be filled in as part of the referral form when referring women to higher level of health facilities • Introduce or enhance facilities’ IT facilities (access to reliable computers, internet and software) to support the implementation of an EMR system • Introduce or strengthen the stillbirth reporting requirements at the policy level by mandating timely notification (e.g. within 48 hours) of stillbirths to designated staff • Invest in building or upgrading investigation or testing facilities within hospitals, such as pathology labs or access to advanced diagnostic equipment • Prompt the HCPs to ask for minimum socio-demographic information and fetal heart rate upon admission with a reminder at the top of the labour room register *Training* • Provide hands-on training to the HCPs on how to use sonography to determine accurate gestational age
Automatic motivation	• Emotional involvement • Mandatory attendance to stillbirth reviews • Stillbirth monitoring by higher levels of the health system	*Environmental restructuring* • Implement a hospital policy that requires mandatory attendance of minimum job roles at stillbirth reviews *Incentivisation* • Provide recognition to hospitals that consistently meet stillbirth reporting requirements and demonstrate improvements in stillbirth monitoring and review processes, as part of wider health system monitoring efforts in India *Persuasion* • Share case studies and personal testimonials from HCPs who turned emotional challenges from stillbirths into contributing to making positive changes in quality of care through participation in stillbirth reviews
Reflective motivation	• Beliefs that stillbirth reviews enable action • Beliefs that there is a low burden of stillbirth • Community’s beliefs about the causes of stillbirth (perceived by HCPs) • Beliefs that stillbirth review can reduce the knowledge gap • Buy-in from wider stakeholders • Opportunities for discussion about the benefits of stillbirth reviews	*Education* • Develop culturally sensitive education materials to address community beliefs about the causes of stillbirth • Provide regular stillbirth data reports comparing the hospital’s stillbirth rates to regional and national averages, emphasising that even a low burden is significant in the community and that each stillbirth review contributes to improving maternal and fetal outcomes • Provide targeted training sessions or workshops to key stakeholders resistant to the stillbirth review process, focusing on the importance of the review, clarifying their roles, and demonstrating how their participation improves maternal and fetal outcomes *Enablement* • Create continuous learning opportunities where HCPs can share knowledge gained from stillbirth reviews with their colleagues and wider health system • Work with traditional health workers, religious or community leaders to help shift community’s perceptions around stillbirth, encouraging community support for stillbirth reviews and participation in data collection *Environmental restructuring* • Establish a multidisciplinary working group that includes representatives from key stakeholder groups across the community, facility, and government (e.g. pathologists, community health workers, obstetricians, nurses, policymakers) *Persuasion* • Conduct regular peer-led discussion sessions where experienced HCPs share their successes and how they navigated challenges during stillbirth reviews • Generate quantitative and qualitative evidence (evaluation) showing how actions taken as a result of stillbirth reviews have led to measurable improvements in pregnancy care
Capability	• Record-keeping skills • Understanding the importance of documentation • Staff trained in how to conduct effective stillbirth reviews	*Enablement* • Provide ongoing support and mentorship programmes where senior staff mentor students and nurses on effective documentation practices *Environmental restructuring* • Implement regular audits of data quality for routine health records and stillbirth documentation *Training* • Develop and deliver specialised training programmes on how to conduct effective stillbirth reviews, including identifying causes of stillbirth, identifying suboptimal care, conducting data analysis and leading discussions to create recommendations of actions • Develop and deliver specialised training programmes on how to organise stillbirth reviews, including identifying eligible stillbirths for review, selecting which stillbirth to prioritise, and gathering the necessary data • Implement mandatory training sessions that focus on improving record-keeping skills

*HCP* healthcare professionals, *EMR* electronic medical records, *IT* information technology

## Discussion

### Principal findings

We examined HCPs’ perspectives of the facilitators and barriers to the implementation and sustainability of facility-based stillbirth reviews in India. Facilitators and barriers related to physical opportunity were the most frequently reported by the HCPs, with a strong emphasis on the need for resources and infrastructure for stillbirth data collection and reporting, as well as improvements in the quality of care. Actionable interventions proposed to address these factors included the standardisation of stillbirth data collection, improving IT infrastructure, and clearly defining and assigning accountability mechanisms.

Participants also highlighted the need for a supportive, blame-free culture and improved engagement across different levels of the health system to improve social opportunities. To address these needs, actionable interventions proposed were implementing regular educational workshops to emphasise the constructive nature of stillbirth reviews, fostering a supportive review culture through senior staff’s proactive demonstration (modelling), and developing culturally sensitive communication strategies to address community concerns about post-mortem examinations.

Regarding automatic and reflective motivation, we recommended creating incentives for staff participation in stillbirth reviews and sharing case studies that demonstrate how stillbirth reviews led to tangible improvements in pregnancy care. Additionally, understanding the community’s beliefs about stillbirth and working with traditional health workers, religious or community leaders to help shift the community’s perceptions around stillbirth was suggested as a means to garner the community’s support for the review process. For improving capability, actionable interventions include mandatory training programmes for effective documentation and review processes, along with ongoing support and mentorship to improve record-keeping skills and review practices.

### Discussion in light of other evidence

The results of this study echo several findings in Willcox et al.’s systematic review of qualitative studies, which looked at determinants of behaviours influencing the implementation of MPDSR in low-and-middle-income countries (LMICs) [9]. Similar to Willcox et al. [9], we found that physical and social opportunities depend on adequate resources (human resources, standard proforma, stillbirth data), regularly scheduled meetings, and opportunities for all levels of the health system to participate in a blame-free environment.

Opportunities to participate without fear of blame should be supported with the ‘education’ intervention function of the Behaviour Change Wheel to prevent leadership teams from misinterpreting stillbirth reviews as a tool for disciplining staff. Previous studies also found that despite the intended goal of stillbirth reviews being to improve the quality of care, the process could be misconstrued when intertwined with other objectives, such as teaching responsibilities [23, 24]. The significance of employing blame-free language and attitudes should also extend beyond the internal dynamics between staff at the facility level; it should be adopted for interactions with women, family members, and the community [25, 26]. In addition, engaging with traditional health workers, religious or community leaders to shift perceptions around stillbirth could foster community engagement in stillbirth reviews and encourage women to participate in interviews with HCPs for data collection [27, 28]. Given that extended family members and fathers often influence decisions in India’s cultural context [26], culturally sensitive communication strategies addressing the community’s common concerns about stillbirth should target a broader audience, not just women [29, 30].

Findings also highlighted that the use of ‘scolding’ language towards women following a stillbirth can discourage them from providing crucial information about their pregnancy. This reluctance not only impedes the collection of necessary data to understand the causes of stillbirth and identify suboptimal care factors, but it also creates greater mistrust between the health system and women. Such mistrust may result in women avoiding healthcare services in future pregnancies, either due to fear of being mistreated or a lack of confidence in the system. This can, in turn, exacerbate health inequalities in maternal and fetal outcomes. Atkins et al. [31] found that 25.4% of parents did not find their care after stillbirth respectful, and parents from middle-income countries were less likely to report respectful care compared with high-income countries. Without urgent efforts to eliminate the disrespectful treatment of parents and raise awareness about stigma, bias, and disrespect around stillbirth, bereaved women and families may face mistreatment under the guise of ‘data collection’ for stillbirth reviews. The International Stillbirth Alliance’s Parent Voices Initiative advocacy toolkit [25] includes training modules for HCPs, covering topics such as the definition of stillbirth, its impact on parents, and the support that health facilities can provide. The toolkit also offers approaches for respectful communication with parents following a stillbirth, along with a checklist for HCPs on how to support parents.

One actionable intervention that can be adopted is a code of conduct ‘charter’ pledging the staff’s commitment to providing a safe environment for all panel members and the community and incorporating this ‘charter’ into the local stillbirth review guidelines. This method has been described as an effective strategy to implement a blame-free culture extending to both HCPs and women [8, 32–34]. The WHO guidelines include a sample meeting code of practice that could serve as a useful reference for developing a ‘charter’ [7, 35]. Establishing a respectful review panel culture would also naturally open opportunities for wider and more willing participation from all levels of the health system and the community.

We identified several interventions to address cultural and procedural barriers to obtaining consent for post-mortem examinations. Supporting HCPs and the community in understanding more about the nature and benefits of post-mortem examinations may help demystify the procedure and alleviate apprehensions. Findings from Lewis et al.’s systematic review [36], also emphasised the importance of clear and compassionate communication and the provision of educational materials tailored to the needs of bereaved families [37, 38]. Additionally, employing ‘community champions’ (also known as community health workers, ambassadors, or peer workers) has been effective in navigating cultural challenges to obtaining consent for post-mortem examinations and improving healthcare utilisation among seldom-heard populations [39–41].

One hospital suggested that exploring an opt-out consent system for post-mortem examinations could help address some barriers observed in the Indian context, similar to the opt-out practice used for HIV testing. However, such a policy may conflict with the primary psychological barrier that parents face, which is the desire to ‘protect’ their baby from perceived unnecessary harm [42–44]. Furthermore, there is currently no literature specifically examining opt-out consent systems for post-mortem examinations. Thus, future community-based research is needed to assess whether an opt-out consent system could be effectively implemented for post-mortem examinations within the Indian context.

Developing culturally sensitive communication materials, such as pamphlets and videos, could address specific concerns related to post-mortem examinations in diverse communities. These materials should reflect local cultural norms and values, as identified in a qualitative study in Delhi, where bereaved families expressed concerns about their child’s disfigurement and wondered what the benefits of the procedure were [45]. Moreover, providing communication training to HCPs can improve their communication skills and confidence in discussing post-mortem examinations with families. The literature supports this approach, highlighting that training and support for staff improve their ability to guide and support bereaved parents and provide respectful care [31, 46–48].

This study found that there are local variations in labelling stillbirth types, especially around antepartum stillbirths. According to United Nations Children’s Fund (UNICEF), stillbirth misclassification can arise from deliberate actions, such as attempts to avoid blame or additional administrative burdens, or inadvertent errors due to unclear guidance on stillbirth definitions [49]. Nevertheless, it is crucial to highlight that excluding antepartum stillbirths from the ‘stillbirth’ category could undermine the perceived importance of these stillbirths among HCPs, as they may view them as less relevant due to the absence of direct care involvement. The study participants emphasised the importance of having clear guidance on the stillbirth definition, as noted in previous studies [3, 10, 50–53]. Additionally, introducing or strengthening stillbirth reporting requirements at the policy level – such as mandating timely notification (e.g. within 48 hours) of stillbirths – will ensure counting and reporting of all stillbirths, regardless of local labelling variations.

Improving the minimum stillbirth data required for reviews can be achieved through a multifaceted approach. First, several hospitals discussed difficulties in getting information from lower-level health facilities where women were receiving care before being referred to their hospitals, and data around antenatal care (which the lower-level health facilities were providing) were often missing from the stillbirth review. To address this, there should be standardised referral and information-sharing systems between different levels of health facilities so data collected during the antenatal period are transferred and stored at the hospital where women give birth [35]. Additionally, involving lower-level health facilities or community health workers in the stillbirth review process could help address gaps in documentation by enabling them to directly contribute the data they collected during antenatal care.

Second, the use of standard proforma for collecting stillbirth data was suggested as a facilitator for many hospitals in this study. The WHO’s ‘Minimum set of perinatal indicators’ form [7, 35] and India’s national stillbirth guidelines ‘Stillbirth review proforma’ [6] would offer a strong foundation for hospitals aiming to standardise their data collection processes. Implementing these proformas should be accompanied by adequate training and ongoing support, including mentorship programmes where senior staff guide students and nurses in effective documentation practices. Additionally, conducting regular audits of data quality for both routine health records and stillbirth documentation could supplement the ‘training’ and ‘enablement’ aspects of the Behaviour Change Wheel intervention functions, ensuring a continuous improvement cycle in data quality and completeness.

Third, health facilities should ensure adequate data storage systems so that all routine health records can be kept safely; not least, this is needed to provide high-quality care, and when data is needed for the stillbirth reviews, it can be pulled out efficiently [9]. Hospitals identified needing adequate IT facilities that enable EMR, which will facilitate their current record-keeping practice. India’s Ayushman Bharat Digital Mission (ABDM), launched in September 2021, may provide a valuable opportunity for health facilities to explore support for EMR implementation [54]. As part of a central sector scheme, ABDM provides funding to state governments for human resources, capacity building, and information, education, and communication activities to facilitate its rollout across all states in India [55]. By leveraging ABDM, health facilities may be better positioned to overcome the financial and operational barriers associated with EMR implementation. However, as McKernan et al. emphasised, the development of EMR will require strong commitment and leadership from the clinical community to be patient-focused throughout its development and implementation [56].

Finally, resources should be available to support improving the quality of fetal heart rate monitoring and gestational age assessment data. As discussed above, this study identified frequent gaps in documentation from referring health facilities, particularly regarding the recording of fetal heart rates prior to transfer. The absence of this information makes it challenging to ascertain whether a stillbirth occurred before or during labour. To address this, it is essential to ensure that referring facilities routinely check and record fetal heart rates and include this information when transferring women to higher-level health facilities. This requires adequate training, availability of equipment, and accountability systems to ensure fetal heart rates are recorded promptly upon a woman’s arrival at any facility, regardless of the onset of labour [57].

This study also identified the use of LMP as an unreliable method for determining gestational age – a common challenge in LMICs identified in the literature [53, 58–62]. To address this, Blencowe et al. [58] recommended improving antenatal care to ensure women receive at least one ultrasound, as per the WHO guidelines [62], and also utilising Artificial Intelligence (AI) to improve the accuracy of ultrasound-based gestational age assessments [63], particularly in settings where trained sonographers are limited or when ultrasound is performed in late pregnancy and accurate dating of gestational age can be more challenging.

All hospitals participating in stillbirth reviews identified that having a regularly scheduled meeting for stillbirth reviews is an important facilitator. However, for hospitals to have such system-level inputs embedded in their clinical practice, significant human resources are required to be committed. For example, additional resources such as extra staff to assist with data collection or organising review meetings and creating protected time for staff to focus on stillbirth reviews without competing with other clinical work demands. Since many hospitals in our study were concerned about the challenges of high workloads preventing them from record-keeping, organising and conducting stillbirth reviews, adequate funding and resources should be provided at the policy level to support hospitals in tackling the logistical challenges of conducting stillbirth reviews.

Staff trained in conducting effective stillbirth reviews was identified as an important capability required for an effective stillbirth review process. This training should address factors that increase motivation, such as the belief that stillbirth reviews enable actions and help reduce knowledge gaps. It should also be tailored to specific roles [9]; for example, staff not directly interacting with patients may need additional support to understand the purpose and benefits of stillbirth reviews. Peer-led discussion sessions, where experienced HCPs share their successes and how they navigated challenges during stillbirth reviews, could be a simple yet effective way to gain broader buy-in from all stakeholders, including community leaders [64].

When stillbirth reviews are conducted well, the findings have the ability to reduce stillbirth rates and other adverse maternal and fetal outcomes by improving all areas of care for all women [7]. In a way, it is a positive cycle because the belief that reviews can lower the burden of stillbirth has the potential to motivate staff to improve the quality of the stillbirth reviews. To embed a culture of accountability in implementing recommendations and drive learning and improvement in the review process, initiatives such as mandatory attendance [8] and developing written documents based on review findings [65] may also be necessary.

### Strengths and limitations

A key strength of our study was the inclusion of FGD participants from hospitals with and without an existing stillbirth review processes so that a wide range of experiences and viewpoints about facilitators and barriers to implementing and sustaining facility-based stillbirth reviews could be captured.

We also identified several common concerns across both groups, providing a more holistic picture of the situation in India regarding facility-based stillbirth reviews. This study highlighted areas for actionable interventions, providing valuable insights into the community, facility and policy level support needed to help hospitals implement national and international initiatives. By employing the COM-B model and the Behaviour Change Wheel intervention functions, we also utilised a theoretical foundation for systematically identifying factors related to opportunity, motivation, and capability and for designing targeted interventions, thereby adding rigour to our recommendations.

One limitation of this study was the potential for selection bias, as the recruitment of FGD participants depended on individuals who chose to engage and could take time off from their clinical duties. This meant that the perspectives of less engaged or more junior staff members who could not arrange cover for their clinical responsibilities might be underrepresented in this study.

Additionally, this study may contain social desirability bias if FGD participants provided responses they believe are expected or acceptable rather than their true opinions. Stillbirth is a difficult and sensitive topic, and fear of blame may have existed among the participants during the FGD. The FGD facilitator (YYB) tried to mitigate this potential bias by acknowledging the sensitivity of the topic and reassuring the participants of the study.

Finally, despite the importance of understanding how local variations in labelling stillbirth types might affect stillbirth statistics or perceptions regarding particular subtypes of stillbirth, this aspect was not explored further. This limitation stemmed from the scope of the study, which did not explicitly focus on this issue. The topic guide for the FGDs was designed to align with the study’s objectives and did not include prompts to explore this matter. Hence, it limited the depth of insights gained on this critical issue.

## Conclusion

This study revealed that the implementation and sustainability of facility-based stillbirth reviews in India can be improved by creating a blame-free environment, ensuring participation from all levels of the health system, and addressing cultural challenges related to perceptions of stillbirth and post-mortem examinations. Facilitators related to physical opportunities, such as having clear stillbirth definitions and collecting minimum stillbirth data, should be supported with targeted training and mentoring to improve HCPs’ capability to utilise these resources effectively. At the policy level, addressing high workload challenges through adequate funding and resources and establishing a monitoring system for stillbirth reviews and reporting would help hospitals embed a culture of accountability, implement recommendations from stillbirth reviews, and drive improvements in the review process. Additionally, reflective motivation – specifically, the beliefs in the benefits of stillbirth reviews for both the facility and the community – was identified as a significant factor in why HCPs would implement and sustain stillbirth reviews despite barriers related to social and physical opportunities.

## Supplementary Information

Supplementary Material 1.

## Data Availability

The datasets used and/or analysed during the current study are available from the corresponding author on reasonable request.

## Abbreviations

ABDM: Ayushman Bharat Digital Mission
AI: Artificial Intelligence
CMO: Chief Medical Officer
COM-B: Capability, opportunity, motivation and behaviour
DPhil: Doctor of Philosophy
EMR: Electronic medical record
FGDs: Focus group discussions
FHS: Fetal heart sound
HCPs: Healthcare professionals
HIV: Human immunodeficiency virus
IT: Information technology
IUDs: Intrauterine deaths
LMICs: Low-and-middle-income countries
LMP: Last menstrual period
MaatHRI: Maternal and perinatal Health Research collaboration, India
MPDSR: Maternal and Perinatal Death Surveillance and Review
NHM: National Health Mission
SOPs: Standard operating procedures
UNICEF: United Nations Children’s Fund
WHO: World Health Organization
