# Supplementary material for: Facilitators and barriers to implementing and sustaining facility-based stillbirth reviews in India: a qualitative study
Source: BMC Pregnancy Childbirth. 2025 Aug 6;25:819. doi: 10.1186/s12884-025-07912-6 (PMC12330080; doi:10.1186/s12884-025-07912-6)
Supplement: Supplementary file 1 — Supplementary Material 1. [file 12884_2025_7912_MOESM1_ESM.docx]

# **Appendix A: Being a facilitator in focus group discussions**

# Reflecting on my experiences as a facilitator in focus group discussions (FGDs) and as a foreign researcher, I wish I had more time to build a greater rapport with the potential participants before the FGDs took place. I am not an Indian. In fact, I am from South Korea, not Britain where my University is located. I come from a different culture and environment than the participants, and I do not speak any languages spoken in India other than English. The only shared position between the participants and I was that we were all there to improve the quality of maternity care and to reduce stillbirths in India. Unfortunately, due to the limitations of my Doctor of Philosophy (DPhil) timeline and funding, I did not have a chance to build trust with the potential participants of the FGD before I travelled to India.

# While I never once felt unwelcomed by the participants, I thought that my position made some participants feel hesitant to share their very honest opinions with me. Perhaps that was why engaging all participants was often challenging at the start of the FGD. During several sessions, I observed that nurses and junior staff (e.g. postgraduate students, resident doctors) tended to contribute less, particularly at the beginning of the FGD. This may be due to existing hierarchies in the hospital and their reluctance to share thoughts in front of senior staff, as well as with me. However, this did not lead me to doubt my decision to conduct FGDs instead of 1:1 interviews. I believe that they might not have engaged with me at all if the discussion had not been held in a group setting. I noticed that some participants who initially did not contribute opened up in the latter half of the FGD and gained the confidence to express their opinions, as they realised that everyone else had been contributing honestly.

I also felt that, at times, since the topic of the FGDs is very sensitive, the conversation around stillbirth and post-mortem examination consent sometimes led to discomfort among participants. This led to discussions occasionally veering off-topic; for example, participants started focusing on specific job roles’ responsibility for stillbirth, and I had to carefully steer the discussion back to the main aim of the FGD. I felt uneasy when this happened, and I started to feel researcher fatigue during the FGD. I was upset that one of the participants was being blamed for issues related to antenatal care provision and late referrals during the FGD I organised and facilitated. As it was my first time in this situation, I did not know how to manage it in the best way. I sought help from my DPhil supervisor and colleagues after this incident. Reflecting back, I believe this event made me stronger. I learned a lot from it, and especially because it happened early on in my fieldwork, it motivated me to practice how to facilitate future FGDs to stay neutral and respectful and complete my planned work effectively. This process also taught me how to manage conflicts in a discussion setting.

One of the cultural challenges I had to navigate was the culture of more than one person talking at the same time. I first noticed this when the recording quality of one of the FGDs was poor because participants were talking over each other, affecting clarity. I soon learned from the Project Manager who was accompanying me to hospitals that this is a common practice in Indian culture and is not considered a negative thing. However, for the sake of recording quality and the need for transcriptions, I started employing clear instructions to participants at the start of the FGD about not speaking when someone else was speaking. This not only improved the quality of the recordings but also encouraged respectful listening among participants.

Despite these challenges, there were also moments of success. In some FGDs, participants were highly motivated and contributed actively, and I think it was because, as more FGDs were completed, I became better at probing at the right time and encouraging responses without making participants feel pressured. This was the moment when I learned that being an outsider is not always going to be a barrier to facilitating FGDs because skills can be learned. I also realised that I could always work on building rapport with the participants through my voice, facial expressions, and respectful reactions, even if I had limited prior interactions with them. The experiences I had as a facilitator certainly taught me that, with open-mindedness and respectful listening, I could overcome initial barriers and connect deeply with people, leading to truly impactful and heartfelt discussions.

# **Appendix B: Topic guide for hospitals that conduct stillbirth reviews**

*Note: The points noted under each question will be used as probe, if required.*

1. What has been working well when conducting stillbirth reviews in your hospital?

- Reflect what practices have been helpful in your hospital when implementing or sustaining stillbirth review process.
- What else do you think could be helpful for your hospital moving forward, to sustain and improve the current stillbirth review process?

1. What has been the challenge(s) when conducting stillbirth reviews in your hospital?

- Can you describe how each challenge affects and which part of the process it affects?
- Can you give an example how you mitigated/overcame these challenges, if possible to mitigate/overcome?

This is the end of the focus group discussion session. Thank you

# **Appendix C: Topic guide for hospitals that do not conduct stillbirth reviews**

*Note: The points noted under each question will be used as probe, if required.*

1. Do you think stillbirth is a problem in the population you look after?

- What do you think can be done to reduce stillbirth?

1. What are your views on conducting stillbirth reviews?

- What do you understand about stillbirth reviews?
- Are you aware of any guidance?

1. How do you define stillbirth in your hospital?

- Do you collect any data around stillbirth?

1. What in your opinion could be an ideal process for stillbirth reviews?

- Who do you think should be invited to participate?
- How do you think the cases should be selected?
- What kind of resources/support should be available?
- What should be the goals/objectives of the reviews?
- How would you use the information you learned from the stillbirth review process?

1. What are your views on implementing a stillbirth review process in your hospital?

- What could be helpful in your hospital if you were to start conducting stillbirth reviews in your hospital?
- Do you foresee any challenges that will arise if you were to start conducting stillbirth reviews in your hospital?
- Can you describe how each challenge will affect and which part of the review process it will affect?
- Can you give an example of how you may mitigate/overcome these challenges, if possible to mitigate/overcome?

This is the end of the focus group discussion session. Thank you.
